# Bipolar disorder with Melnick–Needles syndrome and periventricular nodular heterotopia: two case reports and a review of the literature

**DOI:** 10.1186/s13256-021-03064-1

**Published:** 2021-10-11

**Authors:** Maria Pia Riccio, Giuseppe D’Andrea, Emilia Sarnataro, Maria Marino, Carmela Bravaccio, Umberto Albert

**Affiliations:** 1 Department of Medical and Translational Sciences, Child Neuropsychiatry, Federico II University, Via Pansini 5, Naples, Italy; 2 Department of Biomedical and NeuroMotor Sciences (DIBINEM), University of Bologna, Bologna, Italy; 3 Department of Medicine, Surgery, and Health, University of Trieste, Trieste, Italy

**Keywords:** Bipolar disorder, Melnick–Needles syndrome, Periventricular nodular heterotopias, Case report

## Abstract

**Background:**

Melnick–Needles syndrome and periventricular nodular heterotopia are two usually mutually exclusive phenotypes of F-actin-binding cytoskeletal phosphoprotein Filamin-A mutations. Melnick–Needles syndrome is a rare X-linked condition that is lethal in males and shows great phenotypic variability in affected females. It is caused by mutations in *Filamin-A* gene, which encodes the protein Filamin A. Defects of the human *Filamin-A* gene also cause X-linked periventricular nodular heterotopia, a malformation of neuronal migration characterized by nodules of neurons in inappropriate location adjacent to the walls of the lateral ventricles.

**Case presentation:**

We report on two Caucasian adolescent females, sisters, diagnosed with Melnick–Needles syndrome and bilateral periventricular nodular heterotopia, who developed bipolar disorder and somatic symptoms disorder at a young age. We also present a review of the literature about mental disorders associated with periventricular nodular heterotopia. Our report shows that patients presenting with atypical and heterogeneous psychiatric disease may have an underrecognized anatomical brain abnormality on genetic basis.

**Conclusions:**

We found records of psychiatric disorders associated with periventricular nodular heterotopia; nevertheless, this is the first report of bipolar disorder occurring in individuals with periventricular nodular heterotopia, and the first report of any psychiatric disorder in individuals affected by Melnick–Needles syndrome. In conclusion, this case report may contribute to characterizing the phenotype of this very rare syndrome.

## Background

Melnick–Needles syndrome (MNS, OMIM 309350) is a rare X-linked bone dysplasia, belonging to the otopalatodigital spectrum disorders (OPDSD), with fewer than 70 cases described in the literature (Orphanet Encyclopaedia, May 2015). MNS is essentially limited to females as, when it affects males, it is embryonically or perinatally lethal [1], but survival may be possible due to mosaicism [2]. MNS generally presents with short stature, craniofacial disproportion, prominent supraorbital ridge, exorbitism, oligohypodontia, and micrognathia. Conductive deafness (due to ossicular malformation) and hydronephrosis are also common. Mitral and tricuspid valve prolapse, bowel malrotation, and joint subluxation may be observed. Obstructive sleep apnea syndrome may occur due to mandibular hypoplasia. Complete atrioventricular canal, prune belly syndrome, and omphalocele have been reported in lethally affected males (Orphanet Encyclopaedia, May 2015).

MNS is caused by missense mutation in the gene that encodes the F-actin-binding cytoskeletal phosphoprotein Filamin A (FLNA), a widely expressed protein that interacts with integrin receptors, transmembrane proteins, and several signaling proteins [3]. These interactions are believed to integrate extracellular and cytoplasmic signals with cellular cytoskeleton rearrangements and membrane reshaping [4, 5] and, thus, to allow locomotion of many cell types, including neurons, as shown by the finding of high-level expression in the developing cortex [6].

Defects of the human *Filamin-A* gene also cause X-linked periventricular nodular heterotopia (PNH), a malformation of neuronal migration characterized by nodules of neurons in inappropriate location adjacent to the walls of the lateral ventricles, having failed to migrate away from the embryonic ventricular zone to the developing cerebral cortex [7, 8]. FLNA mutations have been found in a large number of patients with bilateral and symmetrical PNH [7] and represent the main cause for established monogenic forms of PNH accounting for almost all familial X-linked presentations [6]. The most common presentations of PNH are focal seizure disorder, neurological deficits such as dyslexia, developmental delays, and stroke [6, 9–11]. A broad range of neuropsychiatric conditions has been found in patients with PNH independently from *FLNA* gene mutation and type of heterotopia [12].

We aim to describe the cases of two sisters diagnosed with MNS and bilateral PNH, who developed bipolar disorder and somatic symptoms disorder at a young age. We also present a review of the literature about mental disorders associated with PNH. Our report shows that patients presenting with atypical and heterogeneous psychiatric disease may have an underrecognized anatomical brain abnormality on genetic basis. The finding of PNH on magnetic resonance imaging (MRI) of these patients may address more appropriate treatment. Moreover, to the best of our knowledge, this is the first report of psychiatric disease in patients affected by MNS and may contribute to characterizing the phenotype of this very rare syndrome.

## Case presentation

We will describe the cases of two Caucasian females, sisters having a 4-year age difference, diagnosed with MNS and associated PNH, who both required child and adolescent psychiatry assistance at a young age, during adolescence.

The patients inherited from the mother the *FLNA* gene carrying a c.622G>C nucleotide change, that leads to the aminoacidic substitution p.Gly208Arg in the N-terminal domain of Filamin A. The mother was affected as well by MNS, but, due to a milder phenotype, she was diagnosed only after her offspring received the diagnosis.

### Case 1

At the age of 10 years old, the patient came to the attention of a specialized center owing to facial dysmorphism, prominent forehead and ears, bilateral exophthalmos, blue sclera, full cheeks, preauricular fistulas, dental malocclusion, skin dyschromia, valgus knees and elbows, and a history of hip subluxation and left-convex lumbar scoliosis. These findings were consistent with a possible congenital osteodysplasia; thus, she underwent full-body radiography. The radiography showed basicranium thickening, dorsolumbar kyphoscoliosis, bilateral coxa valga, and mild bowing of the radius in both sides. An MRI of the brain was performed, revealing bilateral nodules of periventricular heterotopia (PH), arachnoid cyst of the medial temporal lobe, and volume loss in the hypophysis. She was then referred for genetic counseling: analysis of *FLNA* gene was made, and missense mutation G622C was identified in exon 3 of the gene, leading to diagnosis of MNS.

By the age of 14 years old, she presented with obsessions, repetitive behavior, and paranoid ideation. Later, she developed anxiety symptoms, panic attacks, and episodes of apparent loss of consciousness, accompanied by falls and abnormal movements of the limbs. For the repetitive occurrence of these convulsive-like phenomena, she was admitted to the Neurology Service, and an electroencephalogram (EEG) video study was performed. She was dismissed with the diagnosis of psychogenic non-epileptic seizures (PNES) and received a short treatment with delorazepam. After some months, she presented with a major depressive episode characterized by depressed mood, feelings of worthlessness, psychomotor agitation, social withdrawal, inability to concentrate in studies, and recurrent thoughts of death. For this clinical presentation, she was referred to a private practice psychiatrist and a few months later to an outpatient visit at our Child and Adolescent Neuropsychiatry Service. At the time of the first visit, she had already received treatment with different classes of psychotropic agents, including antipsychotics, antidepressants, and benzodiazepines. During the visit, she presented severe psychomotor agitation, talkativeness, pressured speech, and distractibility, and was referred for outpatient psychodiagnostic. Few weeks later, however, she was urgently admitted to our inpatient for a manic episode with anxiety and reoccurrence of PNES. At the time of admission, she had been prescribed paroxetine 20 mg for a week; she showed emotional hyperreactivity, racing thoughts with derailment, unusual talkativeness and loud voice, disinhibited behavior, and inappropriate social interactions with medical staff. Her hemogram, thyroid, kidney and liver function test, and electrolytes were within normal limits. No alterations were found on EEG during sleep. Electrocardiography (EKG) showed right partial bundle branch block and sinus rhythm. Intelligence quotient (IQ) derived from administration of Wechsler Intelligence Scale for Children (WISC-IV) was 113. She was diagnosed with bipolar disorder and was prescribed valproic acid 900 mg/day and very low dose of risperidone 0.5 mg/day. Later, she was prescribed lithium carbonate at 600 mg/day with good response. Growing up, the patient presented a good compensation for clinical symptoms, thanks to combination of pharmacological treatment and psychotherapy, which also allowed her to follow studies at the university and to lead an independent life.

### Case 2

By the age of 9 years old, the second patient was admitted to inpatient unit of pediatric neurology owing to flaccid paraparesis of lower limbs, lasting 1 week, which was diagnosed as a somatic symptoms disorder. At the age of 10 years old, she underwent the same diagnostic work-up as the first one, as she presented with similar clinical features. The diagnosis of MNS was confirmed by the finding of the missense mutation G622C in exon 3 of the *FLNA* gene. MRI brain scan showed frontoparietal PNH. At the age of 16 years, she was referred to a neuropsychiatric visit at our Child and Adolescent Psychiatry outpatient clinic owing to recurrent depressive episodes that compromised family, social, and school functioning. Full Scale IQ derived from administration of WISC-IV was 105. She presented with depressed mood and referred irritability, recurrent thoughts of death, anhedonia, and apathy. Mood was worse by the morning. She suffered with loss of appetite, fatigue, and central insomnia. She had quit school for a long period and showed difficulties in both concentrating and making decisions. She referred that the recurrent depressive episodes were interrupted by brief periods of elated mood, grandiosity, racing thoughts, increased talkativeness, and decreased need for sleep. She had never been prescribed any psychotropic medication before. She was diagnosed with bipolar disorder, and lithium carbonate was introduced and titrated up to 900 mg/day. The following management of the mood disorder was compromised by lack of compliance to the treatment and adverse reactions to both lithium carbonate and sulfate administration; thus, she went on to develop a disorder with depressive predominant polarity, characterized by school and social withdrawal, and somatic symptoms.

## Discussion and conclusion

MNS is a rare X-linked condition that is lethal in males and shows great phenotypic variability in affected females. It is caused by mutations in *FNLA* gene, which encodes the protein Filamin A. Overall, seven mutations have been found and involve exons 7, 22 (> 90% of cases), and 28 [3, 13, 14]. Our patients exhibited a novel mutation, located in exon 3, which leads to p.Gly208Arg substitution in the calponin homology 2 domain of the actin-binding-domain (ABD) of Filamin A, in a highly evolutionarily conserved residue. This critical non-conservative mutation has been hypothesized to induce both missense substitution and aberrant messenger Ribonucleic Acid (mRNA) splicing, resulting respectively in loss-of-function and gain-of-function of FLNA. This process is thought to have led to the exceptional co-occurrence of PNH (caused by FLNA loss-of-function) and MNS (caused by FLNA gain-of-function), two otherwise mutually exclusive allelic phenotypes [15].

We described the cases of two females affected by MNS and PNH who presented with psychiatric disorders at a young age and were later diagnosed with early-onset bipolar disorder characterized by rapid cycling (not always drug-related), somatic symptoms disorder as epiphenomenon of the mood episode, and different response to mood stabilizers. Neuroimaging studies of subjects with early-onset bipolar disorder found brain anatomical abnormalities in the emotional control network (ECN), such as amygdala volume alterations, and abnormal white-matter microstructure in frontal regions, corpus callosum, and internal capsule, responsible for prefrontal–limbic dysfunctional connectivity [16]. There is no report of PNH associated with early-onset bipolar disorder; however, PNH, especially when there is an underlying FLNA mutation, has been associated with multiple white-matter microstructure alterations, which may be undetectable on common MRI [17].

To date, psychiatric disorders have not been described in association with MNS; searching the literature, we found reports of 33 patients suffering from PNH and psychiatric disorders, mainly reported as single cases. The disorders diagnosed included: psychotic disorders [7, 12, 18–26], depression [12, 27–29], anxiety [23, 30, 31], obsessive-compulsive disorder [23, 32], neurodevelopmental disorders [12, 33, 34], and behavioral disorders [35–38]. Including our patients, there was slight prevalence of females (*n* = 22; 64.7%); mean age at onset of the psychiatric disorder was 26.3 ± 14.2 years, ranging from 8 to 53 years. Intelligence was normal for 40% of subjects (*n* = 14) and borderline for 14.3% (*n* = 5), while the remaining individuals experienced mild-to-severe intellectual disability (ID). Epilepsy was associated with PNH in 54.3% of cases (*n* = 19), and additional MRI findings were reported in 40% of patients (*n* = 14). Underlying genetic mutations were found in 40% of cases (*n* = 14): seven were mutations of FLNA (including our patients), and seven were 22q11.2 deletions. PNH varied from single nodules to bilateral contiguous deposits. Overall, PNH appears associated with a broad range of psychiatric disorders, whose onset presents mainly during adolescence or early adulthood. Additional pharmacologic therapy based on MRI findings may be required to treat the disorder [12, 39]. These reports suggest a link between PNH and psychiatric disorders, but scientific evidence to confirm this association is lacking. Possibly, the neuropsychiatric manifestations result from abnormal brain development on genetic or environmental basis.

Patients with MNS do not usually have PNH, as FLNA-related PNH derives from a loss-of-function mutation, while MNS, as well as the others OPDSD, derives from a gain-of-function mutation of the *FLNA* gene [40]. The structure of FLNA protein and its interaction with actin and various membrane receptors is complex. Possible mutations of FLNA protein are various. This complexity reflects the wide spectrum of phenotypes. In the presented cases, a single mutational event leads to both the missense substitution and the aberrant mRNA splicing of FLNA protein. The c.622G>C substitution caused an aberrant mRNA splicing and also leads to a missense mutation, resulting in loss-of-function of FLNA, associated with the PNH phenotype, and at the same time to a missense mutation, resulting in gain-of-function and in MNS.

Given that psychiatric diseases have not been described in association with MNS or with any of the OPDSDs, a major role in the observed psychiatric disturbances may have been played by bilateral PNH, rather than by MNS per se. In this perspective, psychiatric disorders, more specifically bipolar disorder, may be part of the MNS phenotype when PNH is co-occurring, and thus, MRI scan should be performed in all patients with MNS to detect the possible coexistence of both phenotypes.

In conclusion, this is the first report of bipolar disorder in patients with MNS and PNH. Moreover, this is the first report of psychiatric disorder in a very rare syndrome such as MNS. How the psychiatric features are influenced by the underlying genetic cause of both MNS and PNH remains uncertain. Individuals affected may be at risk for early-onset psychiatric disorders, but further studies are needed to demonstrate the cause of this association. Clinicians should be aware that atypical and heterogeneous psychiatric disorders may be the consequence of an underrecognized anatomic abnormality relying on genetic cause; thus, appropriate diagnostic work-up, including neuroimaging, should be conducted.

## Data Availability

The datasets used and/or analyzed during the current study are available from the corresponding author on reasonable request.

## Abbreviations

MNS: Melnick–Needles syndrome
PNH: Periventricular nodular heterotopia
FLNA: F-actin-binding cytoskeletal phosphoprotein Filamin A
OPDSD: Otopalatodigital spectrum disorders
MRI: Magnetic resonance imaging
PH: Periventricular heterotopias
EEG: Electroencephalogram
PNES: Psychogenic non-epileptic seizures
EKG: Electrocardiography
IQ: Intelligence quotient
WISC-IV: Wechsler Intelligence Scale for Children
ABD: Actin-binding domain
ECN: Emotional control network
ID: Intellectual disability
mRNA: messenger Ribonucleic Acid
